# Metabolic adjustment and regulation of gene expression are essential for increased resistance to severe water deficit and resilience post-stress in soybean

**DOI:** 10.7717/peerj.13118

**Published:** 2022-03-18

**Authors:** Adinan Alves da Silva, Cíntia Oliveira Silva, Vanessa do Rosario Rosa, Michel Filiphy Silva Santos, Kacilda Naomi Kuki, Maximiller Dal-Bianco, Rafael Delmond Bueno, Juraci Alves de Oliveira, Danielle Santos Brito, Alan Carlos Costa, Cleberson Ribeiro

**Affiliations:** 1 Ecophysiology and Plant Productivity Laboratory, Instituto Federal Goiano-Campus Rio Verde, Rio Verde, Goiás, Brazil; 2 Department of General Biology, Universidade Federal de Viçosa, Viçosa, Minas Gerais, Brazil; 3 Department of Agronomy, Universidade Federal de Viçosa, Viçosa, Minas Gerais, Brazil; 4 Department of Biochemistry and Molecular Biology, Universidade Federal de Viçosa, Viçosa, Minas Gerais, Brazil; 5 Ecophysiology and Plant Productivity Laboratory, Instituto Federal Goiano-Campus Rio Verde, Rio Verde, Goiás, Brazil

**Keywords:** Amino acids, Antioxidants, Drought avoidance, Drought tolerance, Osmoregulation, Photosynthesis

## Abstract

**Background:**

Soybean is the main oilseed crop grown in the world; however, drought stress affects its growth and physiology, reducing its yield. The objective of this study was to characterize the physiological, metabolic, and genetic aspects that determine differential resistance to water deficit in soybean genotypes.

**Methods:**

Three soybean genotypes were used in this study, two lineages (L11644 and L13241), and one cultivar (EMBRAPA 48-C48). Plants were grown in pots containing 8 kg of a mixture of soil and sand (2:1) in a greenhouse under sunlight. Soil moisture in the pots was maintained at field capacity until the plants reached the stage of development V4 (third fully expanded leaf). At this time, plants were subjected to three water treatments: Well-Watered (WW) (plants kept under daily irrigation); Water Deficit (WD) (withholding irrigation until plants reached the leaf water potential at predawn of −1.5 ± 0.2 MPa); Rewatered (RW) (plants rehydrated for three days after reached the water deficit). The WW and WD water treatments were evaluated on the eighth day for genotypes L11644 and C48, and on the tenth day for L13241, after interruption of irrigation. For the three genotypes, the treatment RW was evaluated after three days of resumption of irrigation. Physiological, metabolic and gene expression analyses were performed.

**Results:**

Water deficit inhibited growth and gas exchange in all genotypes. The accumulation of osmolytes and the concentrations of chlorophylls and abscisic acid (ABA) were higher in L13241 under stress. The metabolic adjustment of lineages in response to WD occurred in order to accumulate amino acids, carbohydrates, and polyamines in leaves. The expression of genes involved in drought resistance responses was more strongly induced in L13241. In general, rehydration provided recovery of plants to similar conditions of control treatment. Although the C48 and L11644 genotypes have shown some tolerance and resilience responses to severe water deficit, greater efficiency was observed in the L13241 genotype through adjustments in morphological, physiological, genetic and metabolic characteristics that are combined in the same plant. This study contributes to the advancement in the knowledge about the resistance to drought in cultivated plants and provides bases for the genetic improvement of the soybean culture.

## Introduction

Soybean is among the main crops in the world, surpassing 330 million metric tons (MMT) in the 2019/2020 crop year (https://www.statista.com). This leguminous plant has great economic importance for several countries and a relevant role in global food security. However, drought stress in the form of water shortage can negatively impact the crop yield, regardless of the plant development stage (Buezo et al., 2019; Cui et al., 2019).

Plants may change their physiology and metabolism in the presence of environmental stresses. Under water deficit, the main resistance mechanisms involve avoidance and stress tolerance strategies (Polle et al., 2019). Stomatal closure induced by the abscisic acid is an avoidance response to drought and helps to reduce water vapor loss through leaf transpiration, but hinders the diffusion of other gases, restricting the photosynthetic process (Devi & Reddy, 2020; Widuri et al., 2020). To adjust to the low CO_2_ availability in this condition, the photochemical efficiency can be reduced. Furthermore, photosynthesis can be inhibited by non-stomatic factors like those related to the reduction in Rubisco carboxylation rate and ribulose-1,5-bisphosphate regeneration (Pilon et al., 2018). However, the decrease in the photosynthetic rate induced by drought during the vegetative phase of soybean reduces growth and changes the allocation of biomass in the plant, which can reduce grain yield by 40%, besides decreasing seed quality (Thao & Tran, 2012; Krishnan et al., 2014; Mesquita et al., 2020).

Tolerance to water deficit may be achieved through mechanisms that secure cell turgidity and its aptitude to prevent and repair damages (Yang et al., 2020). The increase in antioxidant defenses and osmotic adjustment are the main responses of drought tolerance in cultivated plants (Katuwal, Schwartz & Jespersen, 2020). Thus, to acclimatize to water deficit, plants regulate their gene expression and adjust their physiological and metabolic responses.

The metabolic adjustment in soybean cultivars in response to drought stress can be determined through metabolomics (Feng et al., 2020). The branched-chain amino acids (BCAAs; isoleucine, leucine, and valine), and some metabolites from pentoses phosphates pathway, such as ribose, gluconate, xylose, and xylitol showed increased levels in soybean plants submitted to drought stress (Das, Rushton & Rohila, 2017). Raffinose and galactinol are important osmolytes in cell osmotic adjustment, whose increments in soybean leaves and roots were suggested as a metabolic adjustment response to improve drought tolerance (Tripathi, Rabara & Reese, 2016). Therefore, the identification of responsive key metabolites contributes to elucidate the complex pathway of drought resistance in plants (Chaudhary et al., 2019).

Several genes involved in responses to water deficit in plants have also been uncovered, like those related to Late Embryogenesis Abundant (LEA) proteins, chaperones, antioxidants enzymes, enzymes in the ABA biosynthesis pathway, and transcription factors (Krannich et al., 2015, Tripathi, Rabara & Reese, 2016; Joshi et al., 2016; Takahashi et al., 2018; You et al., 2019). These findings help to understand the responses of plants to deal with drought stress, and additionally are useful for identifying genotypes more suitable for satisfactory growth and productivity even in an unfavorable environment (Batley & David, 2016; Benny et al., 2019).

Studies evaluating physiological, metabolic and genetic characteristics of drought resistance in soybean genotypes have been carried out (Mesquita et al., 2020; Reis et al., 2020). However, there is still a lot to be discovered, since drought resistance in plants depends on a complex set of responses and is influenced by the environment (Manavalan et al., 2009). Thus, all the knowledge generated for soybean plants under water deficit can contribute to the development of cultivars that are more tolerant and resilient to stress. Furthermore, contrasting drought-tolerant and drought-susceptible genotypes has been a simple, but efficient way to identify physiological traits, genes and metabolites that effectively contribute to a more resistant phenotype to stress, which are potential markers for drought tolerance breeding programs (Kumar, Ayachit & Sahoo, 2020; Escalante et al., 2020; Coutinho et al., 2021; Rajarajan et al., 2021).

In the present study, different soybean lineages were subjected to an intense water deficit (1.5 ± 0.2 MPa leaf water potential) to test the hypothesis that acclimatization to stress needs to combine different avoidance and tolerance mechanisms in the same plant. Thus, the aim of this study was to identify morphological and physiological changes, as well as metabolites and genes that determine greater resistance to severe drought in soybean genotypes.

## Materials and Methods

### Plant material, drought stress treatments and experimental design

Three soybean genotypes were used in this study. Seeds of the lineages L11644 and L13241 were obtained from the Germoplasm Bank of the Soybean Improvement Program at the Federal University of Viçosa (Viçosa, Minas Gerais, Brazil) and the seeds of the commercial cultivar EMBRAPA 48 (C48) were obtained by the Brazilian Agricultural Research Corporation (EMBRAPA Soja, Londrina, Brazil). The two lineages were selected from a preliminary trial using 15 soybean genotypes, where they showed physiological contrasting responses (gas exchange and growth) to water deficit. The cultivar C48 was used as a reference for drought-tolerant soybean (Vidal et al., 2012; Mesquita et al., 2020).

Seeds were germinated in a plastic tray containing commercial substrate Bioplant®, and seven days after germination, three seedlings were transplanted to pots containing 8 kg of a mixture of soil and sand (2:1). Each pot was fertilized with 8 g of granulated NPK (20-10-10) before transplanting, and also with 2.8 g of granulated urea 25 days after sowing. Thus, it was avoided that the plants showed symptoms of nutritional deficiency throughout the experiment. Plants were grown for 44 days under sunlight in a greenhouse during the months of September and October, which correspond to the spring season in Brazil. The averages of temperature and relative humidity during the experimental period were 38 °C and 15 °C, 97% and 43%, maximum and minimum, respectively.

All plants were grown in pots with soil under field capacity for 30 days, at which point they reached the stage of development V4 (third fully expanded trifoliate leaf). At this time, the group of control plants (well-watered-WW) was kept under daily irrigation, while another group of plants was submitted to water deficit (water deficit-WD) by suspending irrigation until the plants reached the leaf water potential at predawn of −1.5 ± 0.2 MPa. Upon reaching this water status, plants from treatments WW and WD were subjected to non-destructive assessments, and fully expanded leaves (first and second from apex to base of plant) were harvested for destructive analysis, or immediately frozen in liquid nitrogen. Then, the previously frozen leaves were stored at −80 °C for further analysis (metabolic and gene expression). In addition to the WW and WD treatments, a third group of plants was rehydrated (rewatered-RW) after reached the water deficit of −1.5 ± 0.2 MPa and the soil kept at field capacity for three days; on the fourth day the plants were evaluated and the expanded leaves were harvested as described above.

The experiments were carried out in a completely randomized design, 3 × 3 factorial scheme (3 soybean genotypes × 3 water treatments) and three replicates, totaling 27 pots, each with three plants.

### Shoot and root biometrics

The relative growth rate (RGR) of the shoot was determined according to the methodology proposed by Hunt (1978). The leaf area was measured with an electronic integrator of leaf area (Licor-LI-3100C) and the root length with a ruler graduated in centimeters. The dry matter of the roots and shoot was obtained after drying one plant from each pot, at 65 ± 5 °C in an oven with forced air ventilation until reaching constant weight, and the results were used to determine the root:shoot ratio.

### Plant water status analysis

Leaf water potential (Ψ_w_) was measured in the predawn using a Scholander (Scholander et al., 1965) pressure chamber (model 1000; PMS Instruments, Albany, OR, USA). The Ψ_w_ was monitored daily from the sixth day after the onset of water deficit, when the plants showed evident leaf wilt, until reaching water potential values of −1.5 ± 0.2 MPa. At this time, the relative water content (RWC) was obtained by weighing with an analytical balance, the fresh matter (FM, immediately after collecting the leaf discs), turgid matter (TM, 24 h after soaking the discs in water), and dry matter (DM, oven-dried discs at 60 °C to constant weight) of 10 leaf discs with 0.5 cm of diameter, and calculated using the equation: RWC (%) = (FM − DM)/(TM − DM) × 100 (Barrs & Weatherley, 1962).

### Measurement of gas exchange, chlorophyll *a* fluorescence and chloroplast pigments

Gas-exchange measurements were performed using a portable open-flow infrared gas exchange analyzer (IRGA) system (LI-6400XT, Li-Cor Inc., Lincoln, NE, USA). The measurements were performed from 8:00 h to 11:00 h. Stomatal aperture was maximized using irradiance of 1,000 µmol photons m^−2^ s^−1^ with 10% blue light. The evaluation was performed on the fourth leaf from the apex, with leaf temperature controlled at 25 °C, vapor pressure deficit of approximately 1.0 kPa and chamber CO_2_ concentration of 400 µmol CO_2_ mol^−1^ air. Before data recording, the CO_2_ concentration and the water vapor between the leaf and the reference chamber were automatically matched. The net photosynthetic rate (*A*), the transpiration rate (*E*) and the stomatal conductance (*g*_*s*_) of water vapor were recorded. Instantaneous water use efficiency (iWUE) was calculated as *A*/*E*.

Chlorophyll *a* fluorescence was analyzed on the same leaf where the gas exchange was determined using a fluorometer coupled to the LI-6400XT (IRGA). First, the potential quantum yield of photosystem II (F_v_/F_m_) was obtained in plants adapted for 30 min to the dark. With the plants adapted to light, the effective quantum yield of photosystem II (Φ_II_) and the electron transport rate (ETR) were obtained (Bilger, Schereiber & Bock, 1995). The quantum yield of regulated energy dissipation (Φ_NPQ_) was calculated according to Kramer et al. (2004).

For the quantification of chloroplast pigment content, three leaf discs of five-millimeter diameter from fully-expanded leaves were immersed for 6 h in dimethyl sulfoxide saturated with calcium carbonate, at a temperature of 65 °C. Then, the absorbances of the samples were read in a microplate spectrophotometer (Multiskan GO UV/Vis; Thermo Fisher Scientific, Waltham, MA, USA) at 665, 649, and 480 nm to calculate the concentrations of chlorophyll *a* (C*a* = 12.47A_665.1_ – 3.62A_649.1_), chlorophyll *b* (C*b* = 25.06A_649.1_ − 6.5A_665.1_) and carotenoids (*Car* = (1000A_480_ − 1.29C*a* − 53.78C*b*)/220), respectively (Wellburn, 1994).

### Quantification of abscisic acid (ABA)

Freeze-dried leaf tissue (20 mg) was added to 400 μL of the extracting solution methanol: isopropanol: acetic acid (20/79/1, v/v/v) to extract ABA, according to the methodology described by Müller & Munné-Bosch (2011). Aliquot of the extracts (5 μL) was automatically injected into the LC-MS/MS system (Agilent 1200, Infinity Series), coupled to the triple quadrupole mass spectrometer (QqQ) (Agilent Technologies, 6430, Santa Clara, CA, USA). One standard calibration curve (0.1 to 200 ng) was performed using an abscisic acid standard to obtain absolute quantification.

### Determination of total proteins

Freeze-dried fully expanded leaves (25 mg) were subjected to ethanolic extraction in three sequential steps, in concentrations of 98%, 80%, and 50%. Then, the pellet was resuspended in 400 µL of 0.1 M sodium hydroxide (NaOH) and incubated at 95 °C for 1 h as described by Gibon et al. (2004). After, 3 µL of the supernatant was added to 180 µL of Bradford’s reagent (Bradford, 1976), and the absorbance measured at 595 nm in a microplate spectrophotometer. The concentrations of soluble proteins in the samples were determined using a standard curve of bovine serum albumin (BSA) (0–100 µg mL^−1^).

### Determination of proline and glycine betaine

Proline concentration was determined according to the methodology proposed by Shabnam et al. (2016). Fresh leaf tissue (100 mg) was ground with 1 mL of 3% (w/v) sulfosalicylic acid. Then, 100 µL of the homogenate was added to 200 µL of acidic ninhydrin solution and incubated at 100 °C for 1 h. The absorbance was obtained at 520 nm in a microplate spectrophotometer, and compared to the standard proline curve (0–1.0 μmol mL^−1^).

Glycine betaine content was performed according to Grieve & Grattan (1983), with modifications. Samples (250 mg) of oven-dried leaves (65 °C, 72 h) were ground and shaken at 2,000 × *g* in test tubes with 20 mL of deionized water for 24 h at 20 °C. Extracts were filtered, diluted with 2 N sulfuric acid (H_2_SO_4_) at 1/1 (v/v) and cooled in ice water for 1 h. Cold KI-I_2_ reagent was added and samples stored at 4 °C for 16 h and then centrifuged at 15,000 × *g* for 15 min at 4 °C. The pellet was dissolved in 1,2-dichloroethane and the absorbance was read at 365 nm in a microplate spectrophotometer. The standard curve was performed with commercial betaine (0–100 μg mL^−1^).

### Determination of ascorbate and glutathione

Samples (100 mg) of fully expanded leaves were grinded with 6% (w/v) trichloroacetic acid (TCA), filtered, and then centrifuged at 15,000 × *g* at 4 °C for 5 min to determine the total concentration of ascorbate (Kampfenkel, Montagu & Inzé, 1995). Aliquots of 100 µL of the supernatant were treated with 0.5 mM dithiothreitol (DTT), and 0.02 M sodium phosphate buffer (pH 7.4), at 42 °C for 15 min. Then 0.025% N-ethylmaleimide (w/v), 2.5% TCA (w/v), 8.4% H_3_PO_4_ (v/v), 0.8% 2,2′-dipyridyl (w/v) and 0.3% iron chloride (FeCl_3_) (w/v) were added and incubated at 42 °C for 40 min. After stopping the reaction on ice, the absorbances were measured at 525 nm in a microplate spectrophotometer. The total concentration of ascorbate was determined based on the calibration curve using ascorbic acid standards (0–50 µmol).

Total concentration of glutathione was measured according to Griffith (1980). Fresh leaf samples (100 mg) were homogenized in 0.1 M hydrochloric acid (HCl) and 1 mM ethylenediamine tetra acetic (EDTA), filtered and then centrifuged at 12.000 × *g* at 4 °C for 10 min. Aliquots of 100 μL the supernatant were added to the reaction medium with 25 mM sodium phosphate buffer (pH 7.5), 30 μM NADPH and 0.6 mM DTNB [5,5′ -dithiobis acid (2 -nitrobenzoic)]. After incubation at 30 °C for 5 min, 10 μL of glutathione reductase (50 U mL^−1^) was added and the absorbance was determined at 412 nm in a microplate spectrophotometer. The concentration of glutathione was determined based on the calibration curve using reduced glutathione standards (0–10 µmol).

### Metabolic profile and analysis of gene expression

The analyzes of the metabolic profile and gene expression of the plants were performed only for the lineages 11644 and 13241, according to the results of previous analyses, the most and the least susceptible to water deficit, respectively.

### Metabolite levels measurements

Freeze-dried samples (10 mg) of fully expanded leaves were used for the extraction of metabolites in 1.5 ml of a solution composed of chloroform-methanol-water (1/2.5/1, v/v/v), and ribitol (internal standard, 60 μL/0.2 mg mL^−1^). The samples were kept at 70 °C for 15 min and then centrifuged for 10 min at 14,000 × *g*. An aliquot of 100 μL of the upper phase (polar phase) was collected, transferred to another microtube and kept in a vacuum concentrator (Savant *SpeedVac*; Thermo Scientific, Waltham, MA, USA) for 3 h at room temperature. Then, the aliquots were subjected to derivatization with 40 μL of pyridine containing methoxyamine hydrochloride (20 mg mL^−1^) and 70 μL of the N-Methyl-N-(trimethylsilyl)trifluoroacetamide (Lisec et al., 2006).

The metabolites were analyzed by gas chromatography-mass spectrometry (GC-MS) (Agilent 7890A) and TruTOF® HT TOFMS spectrometer equipped with a 30 m-capillary column (MDN-35). To perform the analysis, 1 μl of the derivatized sample was injected in splitless mode at 230 °C, carried by helium gas in a continuous flow of 2 mL min^−1^. The oven temperature was initially kept constant at 80 °C and then increased by 15 °C min^−1^, until reaching a temperature of 330 °C, which was maintained for 5 min. For relative quantification, metabolite peak areas were normalized to the dry matter of the sample and the peak area of the internal standard ribitol. The results were processed using the TargetSearch program (Cuadros-Inostroza et al., 2009).

### Analysis of gene expression

Approximately 100 mg of freeze-dried leaf samples were added in 750 µL of Trizol® LS reagent (Invitrogen, Waltham, MA, USA) for extraction of total RNA, according to the manufacturer’s protocol. The quantification of the extracted RNA was carried out through the NanoDrop spectrophotometer (ND-1000; Thermo Scientific, Waltham, MA, USA) and the fluorimeter using the Quant-iT™ RiboGreen® RNA kit (Molecular Devices, San Jose, CA, USA). RNA integrity was verified by 1.7% agarose gel electrophoresis. Subsequently, 1 μg of RNA sample was treated with DNAse I Amplification Grade (Thermo Scientific, Waltham, MA, USA) and the synthesis of cDNA was performed using the enzyme M-MLV Reverse Transcriptase (Invitrogen, Carlsbad, CA, USA) following the manufacturer’s protocol.

Real-time polymerase chain reaction (RT-PCR) was used to evaluate the gene expression level. The identification of the genes, the primer sequences, and the encoded proteins are described in supporting “Table S1”. The primers were designed in the Primer Express® software (Applied Biosystems, Foster City, CA, USA) using the complete transcript sequence, obtained in the Phytozome virtual database (Goodstein et al., 2012). The expression of the 60S (Le et al., 2012) and UKN2 (Hu et al., 2009) genes were used as an endogenous control to normalize the expression of the target genes.

PCR analysis were carried out through the Real-Time PCR System (model 7500; Applied Biosystems, Waltham, MA, USA) in 96-well microplates containing 1.0 µL of 10-fold diluted cDNA, 0.6 µM of each primer, and 5 µL of the SYBR Green PCR Master Mix kit (Thermo Fisher Scientific, Waltham, MA, USA). The reactions consisted of the following steps: 95 °C for 10 min and 40 cycles of denaturation at 95 °C for 15 s, followed by annealing and extension at 60 °C for 1 min. The relative expression was normalized by L11644 control treatment using levels of ΔΔCt (2^−ΔΔCt^), according to Livak & Schmittgen (2001).

### Statistical analysis

Data were submitted to test for normality of the residuals (Shapiro-Wilk normality). Data were statistically analyzed using two-way analysis of variance (ANOVA) and tested for significant differences using Tukey’s test and Scott-Knott grouping means. Statistical analyzes were performed using the software RBIO (Bhering, 2017). Graphs were made in SigmaPlot® (Systat Software v.11.0). Analyzes and figures related to metabolic profile were made in the web-based platform “Metaboanalyst” (http://www.metaboanalyst.ca) (Chong, Wishart & Xia, 2019).

## Results

### Plant water status

**Figure 1:**
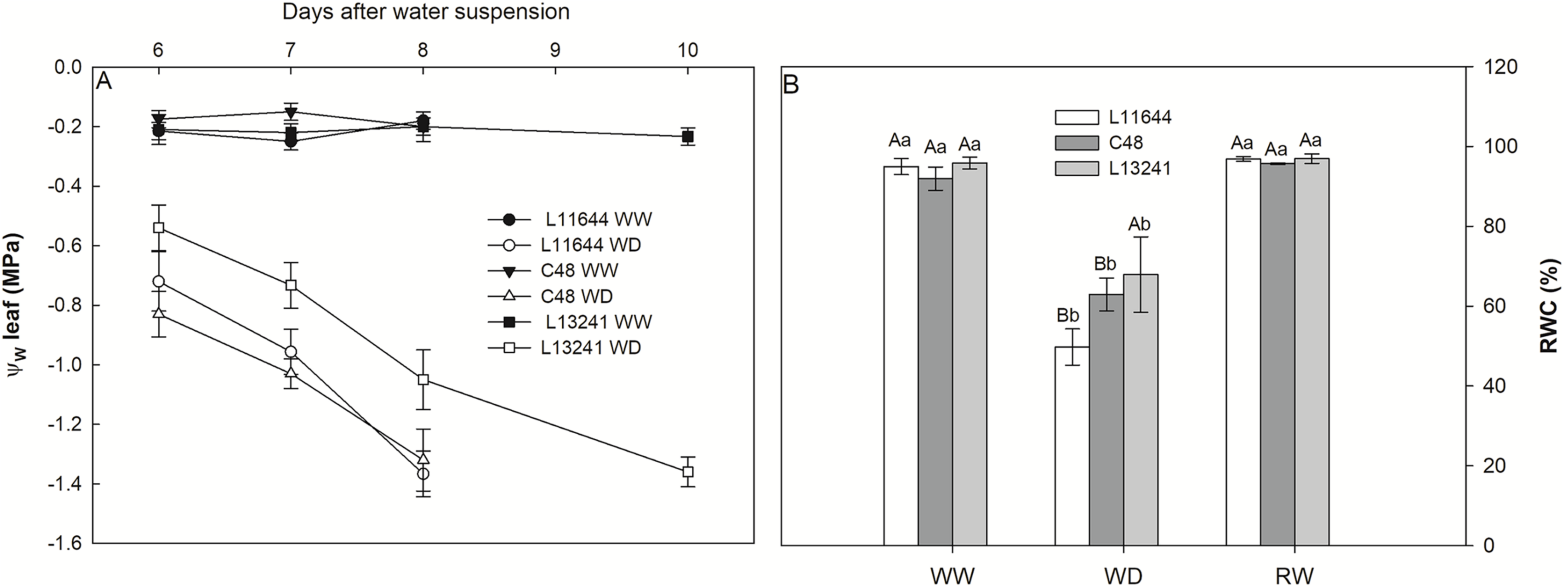
Monitoring the water status of plants. Leaf water potential (Ψ_w_, A) from the sixth to the tenth day after the imposition of water deficit, and relative water content (RWC, B) in three soybean genotypes under three water conditions: WW, well-watered; WD, water deficit; RW, rewatered. Two-way ANOVA for *Genotype* x *Water treatment:* B (*p* = 0.1313). Degrees of freedom: 26. Means followed by different letters, uppercase for genotypes and lowercase for water treatments, differ significantly according to the Tukey’s test. Data are mean of *n* = 3 ± *SEM*.

Genotypes L11644 and C48 reached the threshold of −1.5 MPa leaf water potential (Ψ_w_) on the 8^th^ day after the suspension of irrigation, while L13241 reached said value only on the 10^th^ day (Fig. 1A). The relative water content (RWC) did not differ between the genotypes in the WW and RW treatments (Fig. 1B). However, there was a decrease in RWC for all soybean genotypes under WD, and the L13241 had the smallest reduction, about 30% decrease compared to its control.

### Plant growth and biometrics

**Figure 2:**
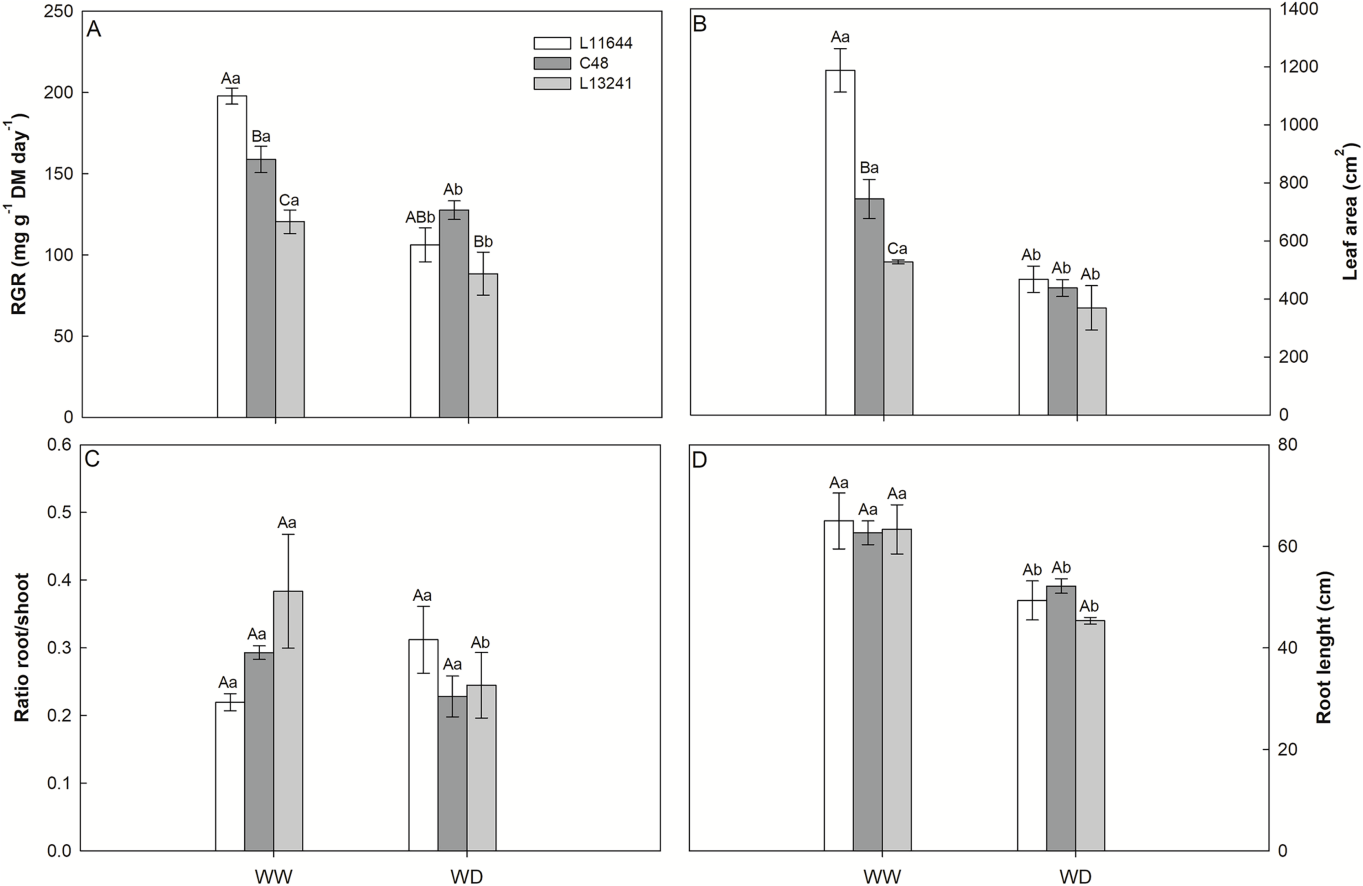
Growth analysis and biometrics. Relative growth rate of shoot (RGR, A), leaf area (B), root/shoot ratio (C) and root length (D) in three soybean genotypes under two water conditions: WW, well-watered; WD, water deficit. Two-way ANOVA for *Genotype* × *Water treatment:* A (*p* = 0.0148); B (*p* = 0.0018); C (*p* = 0.0764); D (*p* = 0.6164). Degrees of freedom: 17. Means followed by different letters, uppercase for genotypes and lowercase for water treatments, differ significantly according to the Tukey’s test. Data are mean of *n* = 3 ± *SEM*.

The relative growth rate (RGR) of the shoot, the leaf area and the root length reduced in all genotypes after WD stress (Figs. 2A, 2B, 2D). However, L13241 showed the smallest reductions in these variables. In the WW treatment, RGR and leaf area were higher in L11644 (Figs. 2A, 2B). The root: shoot ratio did not differ among the genotypes or between water treatments (Fig. 2C).

### Gas exchange, chlorophyll *a* fluorescence and chloroplast pigments

**Figure 3:**
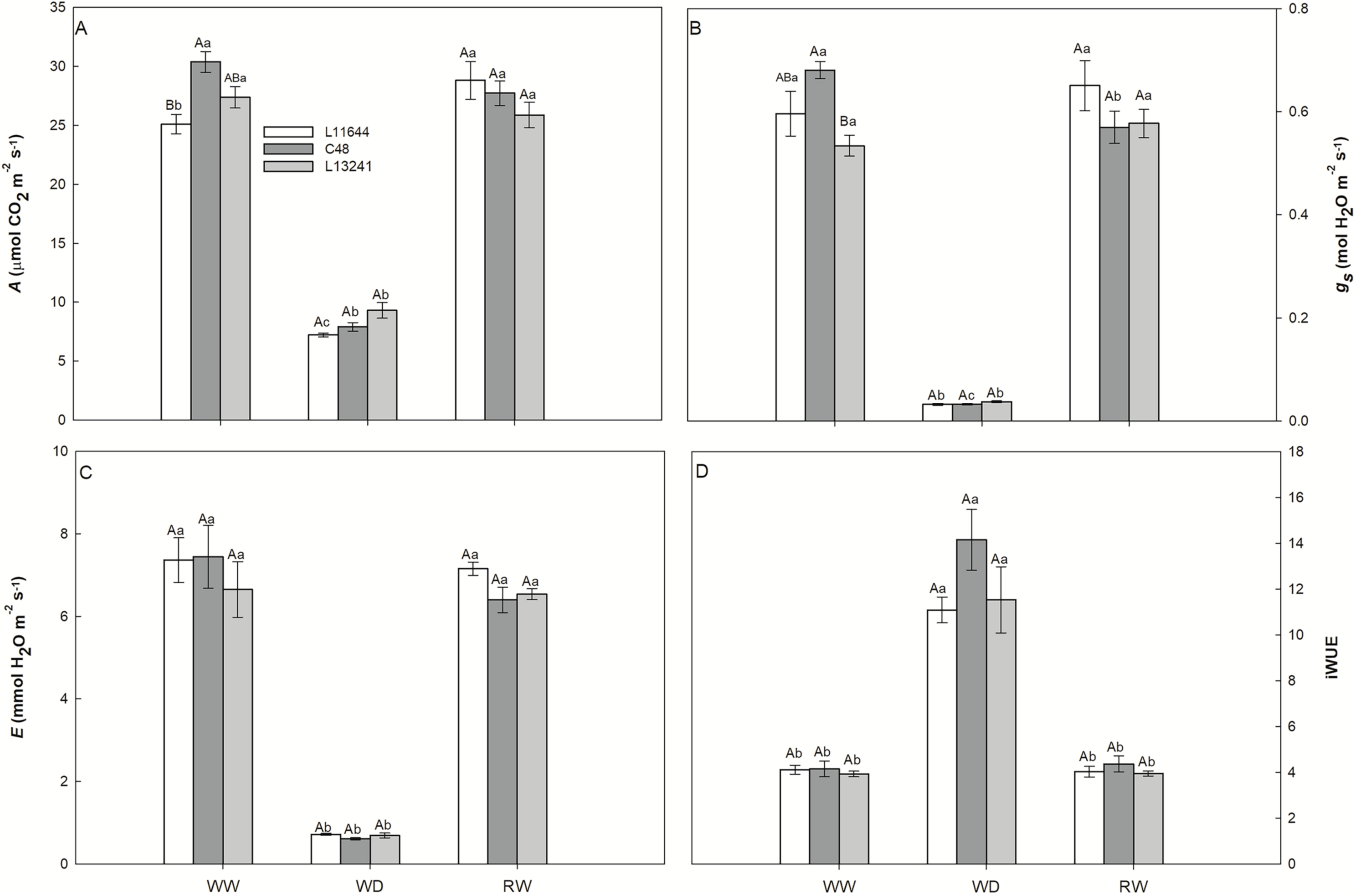
Gas exchange. Net CO_2_ assimilation rate (*A*, A), stomatal conductance (*g_s_*, B), transpiration rate (*E*, C) and instant water use efficiency (iWUE, D) in three soybean genotypes under three water conditions: WW, well-watered; WD, water deficit; RW, rewatered. Two-way ANOVA for *Genotype* x *Water treatment:* A (*p* = 0.0038); B (*p* = 0.0362); C (*p* = 0.6633); D (*p* = 0.1825). Degrees of freedom: 26. Means followed by different letters, uppercase for genotypes and lowercase for water treatments, differ significantly according to the Tukey’s test. Data are mean of *n* = 3 ± *SEM*.

The photosynthetic rate (*A*)_,_ stomatal conductance (*g*_*s*_) and transpiration rate (*E*) decreased in the three soybean genotypes under WD treatment (Figs. 3A–3C). After RW, the plants recovered these variables to the same levels as their respective controls. Instantaneous water use efficiency (iWUE) increased in all genotypes after WD, and there was no difference between them (Fig. 3D).

**Figure 4:**
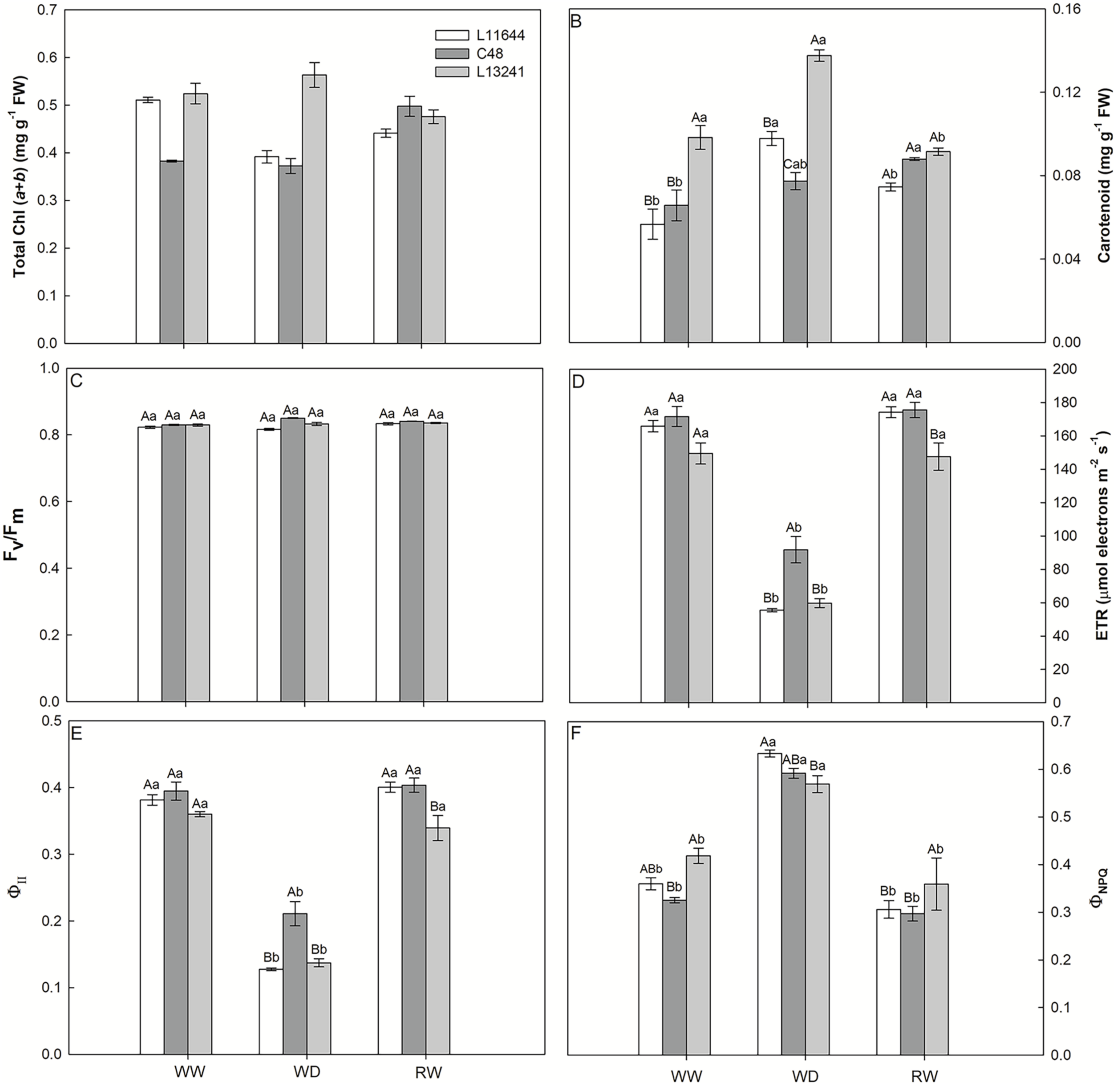
Photosynthesis pigments and chlorophyll *a* fluorescence. Total chlorophyll concentration (Total Chl, A), carotenoid concentration (B), potential quantum yield of photosystem II (F_v_/F_m_, C), electron transport rate (ETR, D), effective quantum yield of photosystem II (Φ_II_, E), and quantum yield of regulated energy dissipation (Φ_NPQ_, F) in three soybean genotypes under three water conditions: WW, well-watered, WD, water deficit; RW, rewatered. Two-way ANOVA for *Genotype* x *Water treatment:* A (*p* < 0.0001); B (*p* < 0.0001); C (*p* = 0.0013); D (*p* = 0.0236); E (*p* = 0.0135); F (*p* = 0.0517). Degrees of freedom: 26. Means followed by different letters, uppercase for genotypes and lowercase for water treatments, differ significantly according to the Tukey’s test. Data are mean of *n* = 3 ± *SEM*.

The concentrations of total chlorophyll and carotenoids were higher in L13241 under WD, compared to other genotypes (Figs. 4A, 4B). In L11644, the total chlorophyll concentration was reduced in WD and remained lower even after RW. There was no variation in the F_v_/F_m_ between genotypes nor treatments (Fig. 4C). ETR and Φ_II_ were reduced in all genotypes under WD (Figs. 4D, 4E), however, C48 showed the highest values. The WD treatment induced the Φ_NPQ_ increases in all soybean genotypes (Fig. 4F). After RW, all plants returned the ETR, Φ_II_, and Φ_NPQ_ values to the same levels of their respective controls.

### Abscisic acid, soluble proteins, osmolytes and antioxidants concentrations

**Figure 5:**
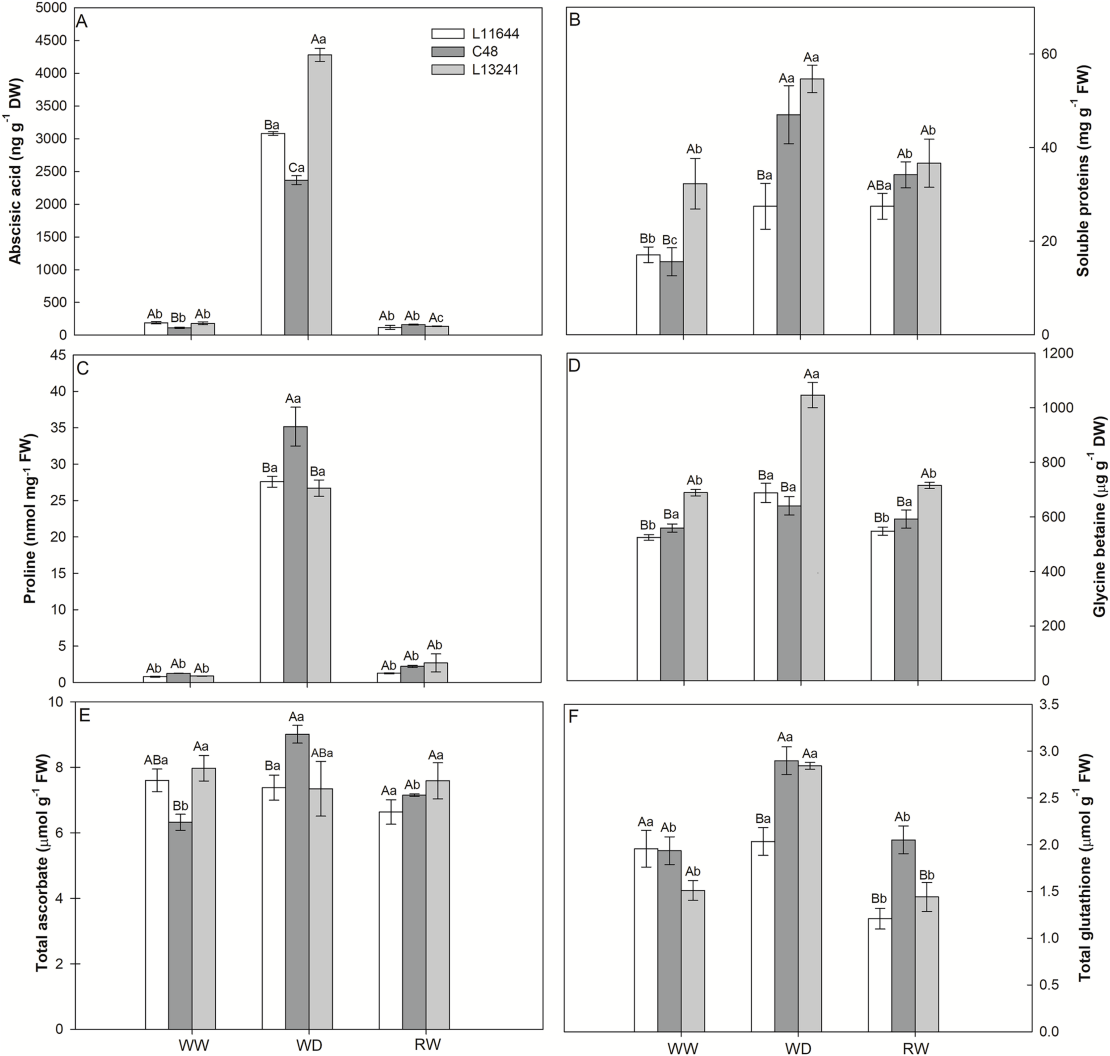
Anti-stress metabolites. Concentrations of abscisic acid (ABA, A), total soluble proteins (B), proline (C), glycine betaine (D), total ascorbate (E) and total glutathione (F) in three soybean genotypes under three water conditions: WW, well-watered, WD, water deficit; RW, rewatered. Two-way ANOVA for *Genotype* × *Water treatment:* A (*p* < 0.0001); B (*p* = 0.0767); C (*p* = 0.0049); D (*p* = 0.0005); E (*p* = 0.0102); F (*p* = 0.0027). Degrees of freedom: 26. Means followed by different letters, uppercase for genotypes and lowercase for water treatments, differ significantly according to the Tukey’s test. Data are mean of *n* = 3 ± *SEM*.

The ABA concentration increased under WD treatment in all soybean genotypes (Fig. 5A). However, the greatest increase was registered for L13241, being 24 times higher than the control. After RW, the ABA concentration returned to values close to those of the control plants.

Similarly, the WD treatment increased the concentration of soluble proteins in all genotypes (Fig. 5B). Only in the L13241 the concentration returned to the same level of control after RW treatment. The proline concentration also increased in all genotypes under WD, but with a greater increase for C48 (Fig. 5C). The WD induced the accumulation of glycine betaine only in L13241, being 52% higher than in the control (Fig. 5D). After RW, the concentrations of proline and glycine betaine returned to similar values of the control treatment (Figs. 5C, 5D).

The WD treatment increased the total ascorbate concentration only in C48, while the total glutathione concentration increased for C48 and L13241 (Figs. 5E, 5F). After RW, the concentrations of total ascorbate and total glutathione returned to the values of their respective controls, except for L11644 whose concentration of total glutathione decreased.

### Relative abundance of metabolites

**Figure 6:**
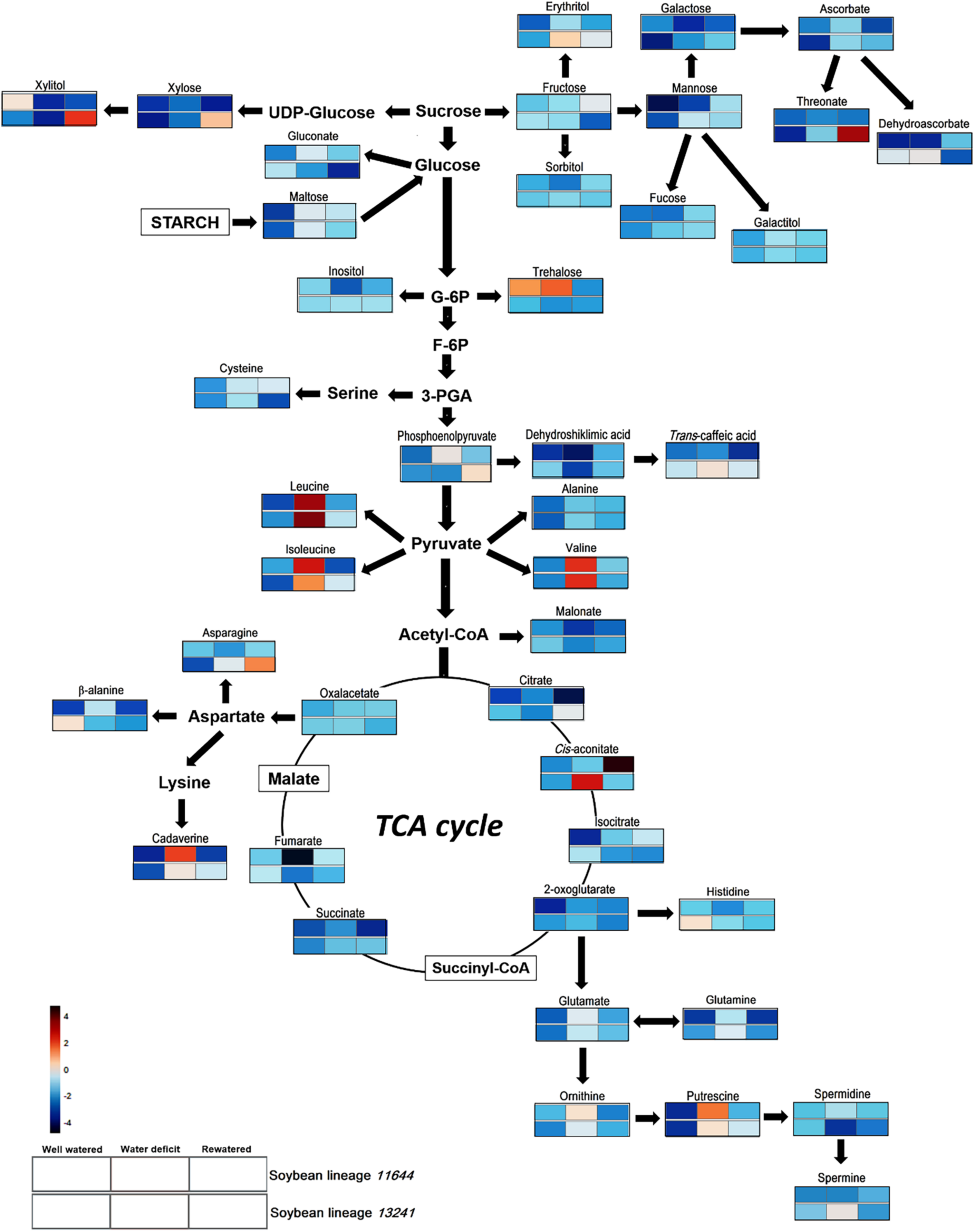
Metabolite profile. Overview of the metabolic pathways representing the relative abundance of metabolites in leaves of two soybean lineages (L11644 and L13241) under three water conditions (well-watered, water deficit and rewatered). Tables with the quantitative data of the metabolites and the results of the statistical analysis are available as supplementary material (Tables S2 and S3).

The analysis of the metabolic profile in the leaves of L11644 and L13241 from all treatments (WW, WD, and RW) allowed identifying a total of 11 amino acids, 15 organic acids, 12 carbohydrates (sugars and polyols) and four polyamines (Table S2, Fig. 6).

There was an increase in the relative abundance of BCAAs (leucine, isoleucine and valine) for both lineages in response to WD. After RW, the relative content of asparagine increased only in the L13241. On the other hand, in WD and RW treatments there was a reduction in the relative abundance of β-alanine and histidine in L13241. Under WD, there was a reduction in the relative abundance of dehydroshikimic acid in both lineages, and fumarate only in L11644. Conversely, the stress increased levels of dehydroascorbate, *trans*-caffeic acid and *cis*-aconitate in L13241, and phosphoenolpyruvate in L11644. After RW, the relative abundance of citrate, phosphoenolpyruvate and threonate increased in L13241 and *cis*-aconitate increased in L11644.

After WD stress, the relative abundance of galactose, xylitol, and inositol decreased in the L11644, however, trehalose increased in this same condition. In L13241, there was an increase of erythritol and mannose after WD, while xylitol decreased. RW promoted increases in the levels of xylitol and xylose in L13241.

Under WD the relative abundance of the cadaverine, putrescine, and spermine increased in L13241. Increases more expressive for cadaverine and putrescine were recorded in L11644. Conversely, the relative abundance of spermidine decreased in L13241 after WD.

### Expression of drought tolerance genes

Under WD, the gene expression analysis showed downregulation of genes related to the proteins from photosynthetic apparatus in both soybean lineages (Table 1). The expression of the genes *RUBISCO ATIVASE* (*RCA*), *CYTOCHROME B6F COMPLEX SUBUNIT 7* (*PetM* cytochrome b6f, electron transport in chloroplasts) and *LIGHT-HARVESTING COMPLEX II* (*LHBC1*, energy absorption in antenna complex) reduced approximately 25, 14, and 100 times in the L11644, and 3, 2, and 1.5 times in L13241, respectively.

**Table 1:** Differential expression of drought tolerance genes.

			*Lineage 11644*			*Lineage 13241*		
	Gene code	Gene name	WW	WD	RW	WW	WD	RW
Photosynthetic process	*Glyma.18g036400*	*RUBISCO ACTIVASE (RCA)*	1.00 ± 0.21a	0.04 ± 0.01b	0.19 ± 0.03a	0.55 ± 0.04a	0.18 ± 0.08b	0.36 ± 0.06a
	*Glyma.07g163600*	*PetM FAMILY OF CYTOCHROME B6F COMPLEX SUBUNIT 7 (PetM)*	1.00 ± 0.26a	0.07 ± 0.00b	0.52 ± 0.06a	0.81 ± 0.09a	0.37 ± 0.15b	0.61 ± 0.10a
	*Glyma.16g165500*	*LIGHT-HARVESTING COMPLEX II CHLOROPHYLL A/B BINDING PROTEIN 1 (LHCB1)*	1.00 ± 0.16a	0.01 ± 0.00b	0.64 ± 0.06a	1.22 ± 0.37a	0.79 ± 0.39b	2.07 ± 0.38a
ABA biosynthesis and signaling	*Glyma.01g43460*	*PROTEIN PHOSPHATASE 2C (PP2C)*	1.00 ± 0.05b	48.50 ± 10.49a	4.60 ± 0.93b	1.69 ± 0.40b	36.36 ± 10.93a	11.70 ± 4.92b
	*Glyma.08g176300*	*9-CIS-EPOXYCAROTENOID DIOXYGENASE NCED3 CHLOROPLASTIC-RELATED (NCED3)*	1.00 ± 0.23b	7.49 ± 0.86a	1.63 ± 0.62b	0.75 ± 0.15b	245.82 ± 119.84a	0.48 ± 0.04b
	*Glyma.11g055700*	*ZEAXANTHIN EPOXIDASE (ZEP. ABA1)*	1.00 ± 0.12a	7.81 ± 1.79a	1.56 ± 0.63a	0.55 ± 0.04a	83.07 ± 40.64a	0.88 ± 0.22a
Antioxidant enzymes	*Glyma.04g248300*	*L-ASCORBATE PEROXIDASE. CHLOROPLASTIC/MITOCHONDRIAL-RELATED*	1.00 ± 0.09a	0.55 ± 0.10a	1.61 ± 0.18a	1.70 ± 0.51a	16.90 ± 8.26a	1.12 ± 0.31a
	*Glyma.12g073100*	*L-ASCORBATE PEROXIDASE 2. CYTOSOLIC*	1.00 ± 0.04a	0.86 ± 0.13a	0.47 ± 0.12a	0.55 ± 0.06a	12.50 ± 6.09a	0.20 ± 0.01a
	*Glyma.02g141800*	*GLUTATHIONE REDUCTASE (NADPH) (GSR, gor)*	1.00 ± 0.31a	0.50 ± 0.08a	0.23 ± 0.03a	0.26 ± 0.02a	1.95 ± 0.76a	0.19 ± 0.02a
Transcription factors	*Glyma.16g02390*	*ABA-INDUCIBLE TRANSCRIPTION FACTOR bHLH TYPE-RELATED (MYC-family)*	1.00 ± 0.20b	30.53 ± 11.51a	0.48 ± 0.11b	0.74 ± 0.09b	414.29 ± 202.67a	0.26 ± 0.06b
	*Glyma.05g35050*	*MYB-LIKE DNA-BINDING PROTEIN*	1.00 ± 0.05a	15.88 ± 3.32a	6.93 ± 1.56a	4.62 ± 0.92a	465.37 ± 227.23a	11.99 ± 4.54a
Osmolyte biosynthesis enzymes	*Glyma.03g137900*	*RAFFINOSE SYNTHASE (E2.4.1.82) (RS)*	1.00 ± 0.05c	1.293 ± 180.31a	1.32 ± 0.45c	0.72 ± 0.08c	76.52 ± 18.39b	0.75 ± 0.20c
	*Glyma.08g288600*	*ALPHA-TREHALOSE-PHOSPHATE SYNTHASE (TPS)*	1.00 ± 0.15a	7.38 ± 0.86a	4.28 ± 1.74a	1.65 ± 0.11a	2.68 ± 0.26a	0.77 ± 0.10a
	*Glyma.19g131500*	*PYRROLINE-5-CARBOXYLATE REDUCTASE (P5CR)*	1.00 ± 0.11b	2.25 ± 0.50a	0.29 ± 0.04b	0.57 ± 0.07b	1.09 ± 0.15a	0.36 ± 0.04b
	*Glyma.20g094500*	*UDP-ALPHA-D-GALACTOSE: MYO-INOSITOL 3-ALPHA-D-GALACTOSYLTRANSFERASE (GALACTINOL SYNTHASE) (GolS)*	1.00 ± 0.17a	8.40 ± 1.11a	2.28 ± 0.73a	1.02 ± 0.35a	64.89 ± 31.72a	2.89 ± 1.00a

**Notes:**

Relative gene expression in leaves of two soybean lineages (L11644 and L13241), in three water conditions: WW, well-watered; WD, water deficit; RW, rewatered.

Two-way ANOVA for *Genotype* x *Water treatment*: *Glyma.18g036400* (*p* = 0.2289); *Glyma.07g163600* (*p* = 0.6824); *Glyma.16g165500* (*p* = 0.5675); *Glyma.01g43460* (*p* = 0.7586); *Glyma.08g176300* (*p* = 0.3984); *Glyma.11g055700* (*p* = 0.4443); *Glyma.04g248300* (*p* = 0.4086); *Glyma.12g073100* (*p* = 0.4074); *Glyma.02g141800* (*p* = 0.2847); *Glyma.16g02390* (*p* = 0.4343); *Glyma.05g35050* (*p* = 0.4108); *Glyma.03g137900* (*p* = 0.0017); *Glyma.08g288600* (*p* = 0.2575); *Glyma.19g131500* (*p* = 0.3918); *Glyma.20g094500* (*p* = 0.4794). Degrees of freedom = 17. Different letters denote significant differences among the treatments, according to the Scott-Knott grouping means. Data are mean of *n* = 3 ± *SEM*.

The *PROTEIN PHOSPHATASE 2C* (*PP2C*) gene, which encodes a protein in the abscisic acid (ABA) signaling pathway, showed a significant increase in response to the WD in both lineages (Table 1). Similar results were observed for the genes *9-CIS-EPOXYCAROTENOID DIOXYGENASE* (*NCED3*), and *ZEAXANTHIN EPOXIDASE* (*ZEP*), both enzymes in the ABA biosynthesis pathway. The largest increases in the relative expression of *NCED3* and *ZEP* genes were found in L13241, about 32 and 10 times higher than in L11644. Despite this, changes in the expression of the *ZEP* gene were not statistically significant (Table 1).

The relative expression of genes encoding antioxidant enzymes varied between the soybean lineages, although there were no statistical differences. In L13241 under WD, the gene expression of L-ascorbate peroxidase enzymes in the organellar (*APX CHLOROPLAST/MITOCHONDRIAL*) and cytosolic (*APX CYTOSOLIC*) isoforms increased 9 and 22 times, respectively, and the expression of glutathione reductase NADPH dependent (GSR) increased 7.5 times. Conversely, lower values for relative expression of these genes were observed in L11644 under WD treatment (Table 1).

The relative expression of genes encoding transcription factors (TF) responsive to drought stress in the MYC (*ABA-INDUCIBLE TRANSCRIPTION FACTOR bHLH TYPE-RELATED*) and MYB (*MYB-LIKE DNA-BINDING PROTEIN)* families increased in both soybean lineages under WD. However, in L13241 the increments were, respectively, 13, and 29 times higher compared to L11644. Nevertheless, the differences in *MYB* gene expression were not statistically significant (Table 1).

The gene expression for enzymes in the biosynthesis of raffinose (*RAFFINOSE SYNTHASE-RS*) and proline (*PYRROLINE-5-CARBOXYLATE REDUCTASE/P5CR*) showed increases in relative expression in both lineages, however greater increases were observed in L11644, mainly for *RS* (Table 1). The expression of TREHALOSE-PHOSPHATE SYNTHASE (*TPS*) and *GALACTINOL SYNTHASE* (*GolS*) genes, which encode key enzymes in the trehalose and galactinol biosynthesis pathways, increased in both soybean lineages after WD stress, although without statistical differences among the treatments. However, the highest relative expression of the *TPS* was observed in L11644 and *GolS* in L13241, respectively.

In general, the level of relative gene expression returned to the same level as the WW treatment after the rehydration (RW) of the plants (Table 1).

## Discussion

### Soybean genotypes performance under severe water deficit

Differential tolerance to water deficit in plants depends on several factors, such as genotype, stage of development, intensity and duration of stress. In this study, we compared differential drought tolerance responses among three soybean genotypes, the cultivar EMBRAPA 48 (C48), already reported as tolerant to stress (Mesquita et al., 2020), and the lineages L11644 and L13241. Interestingly, we observed that L13241 showed better drought tolerance responses than those presented by C48. This was demonstrated by the results of the analysis of the water status, gas exchange, chlorophyll *a* fluorescence, abscisic acid content and concentration of osmolytes and antioxidant metabolites. Conversely, L11644 seems to be more susceptible to the stress. Subsequently, analysis of the metabolic profile and expression of genes responsive to drought corroborated the greater tolerance of L13241 compared to L11644.

### Morphological traits and stress avoidance

Phenotypic characteristics of the L13241, such as lower RGR and leaf area in WW and WD treatments, possible contributed to the improvement of water conservation through growth of the root system and reduction in the transpiration surface, resulting in greater hydration of its tissues. These aspects contributed to postponing water deficiency in this lineage (Fig. 1A). The smaller leaf area of L13241 in WW treatment is an important constitutive characteristic for the identification of soybean cultivars more tolerant to drought (He et al., 2017). Taken together, root and shoot morphological characteristics are important components of the drought avoidance strategy in herbaceous plants (Kooyers, 2015).

### ABA biosynthesis was essential for saving water in soybean genotypes under water deficit

The increase in ABA synthesis also contributed to the maintenance of water status in L13241 under WD treatment (Fig. 5A). This hormone is directly involved in resistance to drought by inducing stomatal closure (Iovieno et al., 2016), either in response to reduced humidity in the environment (Sussmilch & McAdam, 2017) or in the level of hydration in plant tissues (Sack, John & Buckley, 2018). Although the ABA synthesis time-course has not been carried out in this study, the increased expression of the genes *PP2C*, *NCED3* and ZEP in L13241, which encode proteins involved with ABA biosynthesis and signaling (Table 1), demonstrated the important role of this hormone in soybean in response to the water shortage. Such responses resulted in a high content of ABA in leaves of soybean genotypes, mainly in L13241, and consequent reduction in stomatal conductance (*gs*), saving the water available to the plant (Cochetel et al., 2020).

### The decrease in *gs* similarly affects the gas exchange of soybean genotypes

The reduction in the *g*_*s*_ in soybean genotypes denotes a diffusive restriction in the photosynthetic process. This mechanism is common in soybean plants grown under water restriction due to the advantage of reducing leaf transpiration (Gorthi, Volenec & Welp, 2019), but as seen in the present study, this response limits photosynthesis (Fig. 3A). The similarity of the responses observed in plants with greater or less susceptibility to stress demonstrated that gas exchange parameters were not reliable to compare drought tolerance levels between the soybean genotypes (Fig. 3).

In addition to the diffusive restriction on the entry of CO_2_ in the mesophyll, the analysis of gene expression (Table 1) showed negative regulation of photosynthesis by drought also at the transcriptional level (Nouri, Moumeni & Komatsu, 2015). Thus, *RCA*, *PetM* and *LHBC1* genes, which encode proteins of the photosynthetic process, also had their expressions reduced by the water deficit in the lineages 13241 and 11644, but more intensely in the genotype susceptible to stress. Indeed, even after rehydration the L11644 did not recover the expression of the *RCA* gene to the same level as the WW treatment. According to Rivas et al. (2016), after the resumption of irrigation, water deficit tolerant cowpea genotypes are likely to recover their maximum photosynthetic capacity faster than those more sensitive.

The instantaneous water use efficiency (iWUE) is an important parameter for the selection of soybean cultivars more tolerant to drought, because it demonstrates water savings capable of increasing crop yield under stress (Gorthi, Volenec & Welp, 2019). In the present study, the iWUE increased in all soybean genotypes after WD, highlighting the C48 (Fig. 3D). However, analysis of the iWUE only at the leaf level may not be efficient to differentiate tolerance between genotypes, being recommended to carry out analysis of the isotopic carbon composition (δ^13^C) (Medrano et al., 2015; Gorthi, Volenec & Welp, 2019).

### The absorption and use of light energy were adjusted to avoid damage to the plants’ photosynthetic machinery

Despite the photosynthetic limitation induced by the WD treatment, F_v_/F_m_ values close to 0.8 (Fig. 4C) indicated the physical stability of the photosynthetic apparatus of soybean genotypes (Jumrani & Bhatia, 2019). On the other hand, ETR and Φ_II_ are more susceptible to drought and reduced in all soybean genotypes (Figs. 4D, 4E), as a possible preventive response against photooxidative damage to the photosynthetic machinery of plants (Gururani, Venkatesh & Tran, 2015; Yamori, 2016). Inversely to photochemical efficiency, the fraction of light energy dissipated in the form of heat by the xanthophyll cycle in photosystem II, represented by the variable Φ_NPQ_ (Fig. 4F) increased in the WD treatment. This mechanism is activated in response to drought and acts on the photoprotection of plants (Gomes et al., 2020). The reduction in Φ_NPQ_ after RW treatment to levels like th control denotes the resumption of photochemistry as the main pathway for the dissipation of light energy in plants. The recovery of ETR and Φ_II_ in the plants of the RW treatment support this statement (Efeoğlu, Ekmekçi & Çiçek, 2009).

Even with the highest concentrations of chlorophylls and carotenoids under WD (Figs. 4A, 4B), the photosynthetic apparatus of L13241 was not able to tolerate drought. This may be due to the intensity of the stress applied in this study, which affected different steps of photosynthesis causing a restriction in the diffusion of gases and inhibition of PSII. However, higher levels of photosynthetic pigments may improve drought tolerance, especially carotenoids, which can act as antioxidants (Talebi et al., 2013; Abbasi et al., 2014; Khoyerdi, Shamshiri & Estaji, 2016; Mibei, 2017).

### Osmotic adjustment was the main mechanism of metabolic regulation in response to severe water deficit in soybean

The increase in the concentration of osmoregulatory metabolites after WD treatment (Figs. 5C, 5D) in soybean genotypes suggests osmotic adjustment, which acts as an important drought tolerance mechanism (Katuwal, Schwartz & Jespersen, 2020). Accumulation of proline and glycine-betaine is a common response in plants exposed to water restriction, condition in which these metabolites act as cell osmoregulators and osmoprotectants (Ahmad, Lim & Kwon, 2013; Wani et al., 2013; Kaur & Asthir, 2015). Thus, the high accumulation of proline and glycine-betaine in L13241 under WD treatment may have contributed to the lower reduction in its water status (Fig. 1) and growth (Fig. 2) (Singh et al., 2015; Basu et al., 2016).

Proline may also be important for plant recovery after water deficit because it is metabolized in mitochondria and provides ATP to repair damage and resumption growth (Araújo et al., 2013). This would explain the drastic reduction in proline concentration in soybean genotypes after RW treatment. The increases in proline concentration coincided with the increase in the relative expression of the *P5CR* gene (Table 1) that encodes pyrroline-5-carboxylate reductase, a key enzyme in the biosynthesis of this metabolite (Liu et al., 2019).

The metabolic adjustment in the soybean lineages exposed to WD could be seen by the accumulation of defense metabolites against stress, especially osmoprotectants and antioxidants (Figs. 5 and 6). Indeed, sugars and polyols perform these functions in plants under drought (Singh et al., 2015; Zulfiqar, Akram & Ashraf, 2020). The increase in the abundance of trehalose in L11644 and erythritol in L13241 under WD denotes that the accumulation of osmoprotectants occurs in response to stress, regardless of the greater or lesser susceptibility of the genotype (Li et al., 2019; You et al., 2019). Increments in the relative expression of the genes *RS*, *TPS*, *P5CR*, and *GolS*, from the raffinose, trehalose, proline, and galactinol biosynthesis pathways, respectively, corroborate this statement (Table 1 and Fig. 6).

Trehalose and raffinose contribute to the osmotic adjustment and protection of cell membranes in plants under water deficit, in addition to stomatal control and improved water use efficiency (Sengupta et al., 2015; Kosar et al., 2019). Erythritol is a polyol responsive to water deficit and may accumulate even in plants under moderate stress (Fàbregas & Fernie, 2019). In this study, the most susceptible lineage (11644) seems to manage its metabolism and gene expression towards the accumulation of sugars, while the most tolerant (13241) showed a tendency to accumulate polyols. In both cases, these responses would lead to an osmotic adjustment of soybean plants.

Increases in the relative abundance of amino acids in legumes under drought has been described as an important component of cell osmoprotection and improved productivity (Goufo et al., 2017). Abundance of isoleucine, leucine and valine, known as the branched-chain amino acids (BCAA), is a common response in crops subjected to water deficit (Michaletti et al., 2018; You et al., 2019; Coutinho et al., 2019). As expected, BCAA increased in both soybean lineages in this study. BCAAs may serve as a source of energy when the availability of sugars in cells is reduced, and their catabolism may contribute to greater drought tolerance (Pires et al., 2016; Fàbregas & Fernie, 2019). The accumulation of aconitate, observed in L13241, may also function as a reserve energy pool in the form of the tricarboxylic acid (Igamberdiev & Eprintsev, 2016).

Variations in the abundance of glutamate and ornithine in the soybean lineages of this study may be related to the regulation of proline and polyamine levels in plants (Majumdar et al., 2016; Paschalidis et al., 2019). Putrescine (Put), spermidine (Spd), spermine (Spm) and cadaverine (Cad), metabolites known as polyamines, act on drought tolerance through the control of stomatal opening, osmotic adjustment and antioxidant defenses (Nahar et al., 2017; Handa, Fatima & Mattoo, 2018; Chen et al., 2019). Thus, soybean lineages accumulated different types of polyamines as a possible defense mechanism against water deficit stress.

High endogenous levels of polyamines, mainly Spm, as noted in L13241, have been related to greater elimination of ROS, stability of cell membranes and activation of antioxidant enzymes in tomato plants, resulting in greater tolerance to dehydration (Sánchez-Rodríguez, Romero & Ruiz, 2016). On the other hand, there was a reduction in the abundance of some metabolites in both lineages after the water deficit (Fig. 6). The reduction in photosynthesis and cell respiration due to severe drought may affect the biosynthesis of several compounds, such as carbohydrates, organic acids, and secondary metabolites (Das, Rushton & Rohila, 2017). This helps to explain this response, but also, it may be a consequence of cellular metabolism adjusting to stress.

### Differential tolerance to severe water deficit in soybean was also determined by antioxidant defenses

Antioxidant defenses are part of the drought tolerance mechanisms, attenuating cellular oxidative stress (Katuwal, Schwartz & Jespersen, 2020). The highest levels of ascorbate (AA) and glutathione (GSH) observed (Figs. 5E, 5F) in L13241, but mainly in C48 under WD treatment may indicate the role of these metabolites in the direct or indirect elimination of ROS, preventing oxidative damages (Hasanuzzaman et al., 2017). Thus, accumulation of antioxidants may be a tolerance metabolic marker in soybean genotypes under severe drought.

The lack of alteration in the AA (Fig. 5A) content in L13241 is according to the metabolic profile result (Fig. 6). Concomitantly, the increase in the relative abundance of dehydroascorbate (DHA) (Fig. 6) corroborates the increase in the expression of genes encoding chloroplast and cytosolic isoforms of the ascorbate peroxidase enzyme (APX), which oxidize AA to DHA during the elimination of ROS. Similar result was observed for the expression of the gene that codes for the NADPH-dependent glutathione reductase (*GSR*) enzyme (Table 1). Therefore, we suggest that the ascorbate-glutathione cycle is one of the mechanisms for L13241 to tolerate oxidative stress triggered by water deficit (Sarker & Oba, 2018). The positive regulation of genes coding for the APX and GR enzymes has been related to a greater capacity of different plant species to tolerate drought (Pan et al., 2016; Ren et al., 2016).

### Regulation of gene expression is an important mechanism for a more drought-tolerant phenotype

Increases in the gene expression for antioxidant enzyme and drought-responsive transcription factor (TF), suggest the accumulation of these regulatory proteins in L13241 (Table 1). This reasoning is reinforced by the increase in the concentration of soluble proteins (Fig. 5B). Protein increments may be related to accumulation of molecular chaperones, osmotins and TFs (Wang et al., 2016; Cao et al., 2017; Shan et al., 2018), that improve drought tolerance in plants. Conversely, after RW the expression of these genes reduced. Consequently, the concentrations of proteins related also returned to baseline levels (Hao et al., 2015).

Several TFs regulate the expression of target genes inducing the production defense proteins against drought stress (Leng & Zhao, 2020). Increments in the relative expression of genes encoding FTs of the MYC and MYB types are responsive to the dehydration of soybean plants, suggesting their role in drought tolerance (Belamkar et al., 2014; Tripathi, Rabara & Reese, 2016). Although the greatest increases were recorded in the L13241, the results indicate that drought-responsive FTs are induced in both lineages. However, the tolerance will depend on a complex set of morphological, physiological, biochemical and metabolic characteristics in soybean plants (Dubey et al., 2019).

### Post-stress resilience helps to identify soybean genotypes with resistance responses

The return of irrigation restored gene expression to levels like those of the control treatment in the two soybean genotypes. According to Chen et al. (2013), genes responsive to environmental conditions may improve soybean tolerance to water deficit and promote plant growth and recovery after reestablishing adequate water status. Conversely, even after RW, many metabolites maintained high levels in L13241. According to Perlikowski et al. (2016), this response may ensure the better physiological performance of the plant until its full recovery after stress.

## Conclusions

In conclusion, our study demonstrated the joint action of different strategies to resist water deficit in soybean genotypes. The results of the variables WUE, ETR and φPSII denote greater fitness of the photosynthetic process of the C48 genotype to face the water deficit. Lineage 13241 showed avoidance responses to stress, such as smaller leaf area and a higher concentration of ABA in leaves, which contributed to slower water loss and maintenance of higher RWC. The metabolic adjustment in response to stress was mainly directed towards cellular osmoregulation and osmoprotection, highlighting the role of branched-chain amino acids, sugar trehalose and the polyamines putrescine and cadaverine. The expression of genes involved in the drought response in plants, such as ABA biosynthesis, antioxidant defenses and stress-responsive transcription factors was induced more intensely in the lineage13241. These results corroborated the differential response to drought stress between the lineages, being L11644 more susceptible, while L13241 is more tolerant.

In general, post-stress rehydration resulted in the recovery of the plants, showing that even after severe water deficit, with the return of adequate conditions the soybean plants can recover to a state of water and metabolic balance. This study provides a set of relevant information for the genetic improvement of the soybean crop, regarding drought resistance. Additionally, also contributes to the advance in the knowledge on drought resistance in cultivated plants, which integrates physiological, metabolic, and genetic bases.

## Supplemental Information

10.7717/peerj.13118/supp-1 Supplemental Information 1 Primer sequences of the genes analyzed *via* Real-time PCR.

10.7717/peerj.13118/supp-2 Supplemental Information 2 Raw dataset for calculations and statistical analysis. The data are values of the repetitions of each treatment (soybean genotypes x soil water conditions) for the results of physiological, metabolic and gene expression analysis. Data were used for calculations (gene expression analysis) and statistical analysis.

10.7717/peerj.13118/supp-3 Supplemental Information 3 Graphical abstract of the main strategies of the water deficit tolerant soybean genotype.

## Additional Information and Declarations

### Data Availability

The following information was supplied regarding data availability: The raw data are available in the Supplemental Files.
