# Supplementary material for: Metabolic adjustment and regulation of gene expression are essential for increased resistance to severe water deficit and resilience post-stress in soybean
Source: PeerJ. 2022 Mar 18;10:e13118. doi: 10.7717/peerj.13118 (PMC8935993; doi:10.7717/peerj.13118)
Supplement: Supplemental Information 1 [file peerj-10-13118-s001.docx]

**Supplementary material**

**Table S1.** Primers sequences of the genes analyzed via Real-time PCR.

| **Gene** | **Forward** | **Reverse** |
| --- | --- | --- |
| *Glyma.16g02390* | AAGCCAATGGAGCCAAGTCA | GTGCCTCCATTTTCTGATTCACT |
| *Glyma.05g35050* | GGGCCCATGATCCAATGAG | TGGATAAGCAAACACGCTATGAGT |
| *Glyma. 08g176300* | TGGTGTCGACGGTGATGAAG | TCTGCAGCTCCGATTTCCA |
| *Glyma.11g055700* | GGATGGTGCTCTTATGTTGCAAT | ACAACGCTTGCTCATGTCAACT |
| *Glyma.01g43460* | GCTATCGCTCACTTTATTTTATTTGCT | CGTGAAAAGTTAGAAGAGAGAATGAAATT |
| *Glyma.08g288600* | GTGCCACCGAGAGATTTCGT | GGTGCCCCCATTGTTCAGT |
| *Glyma.19g131500* | CGGTCGAATTTTCAGTCACTATTATG | CGAAAAGACTGTTATGGCCAAAC |
| *Glyma.03g137900* | TATCATGTAGTGGAGAAAAAGGGTTGT | GACTTCCCCACCCCATGACT |
| *Glyma.20g094500* | GTTCTCTTTTTCTGGTGCTGATTCT | CACCACCGACACCGTTACAG |
| *Glyma.04g248300* | TCACTAGCAATTGACGGAAAGATG | AGAAGCACATATGGTCAGCAGAGA |
| *Glyma.12g073100* | GCCACTGTGGGTTCCTCAAA | AACCCAACTCCAATCATCATCAC |
| *Glyma.02g141800* | TTCAGAACGATCTTCAGCTACTGAGT | CCAGAGAAGTATGACACACGACCTT |
| *Glyma.07g163600* | AAGGTGGTGAGCTCATTGAAATATC | GCAGCATTGCATGTTGAAGAG |
| *Glyma.16g165500* | AATTGTGACAGGAAAGGGACCTT | CATAGGCCCAGGCATTATTGTT |
| *Glyma.18g036400* | GGCCACCAAATCATAAACATTACA | GGTTTGGGTGCTCATTTCTTTT |

**Table S2.** Leaf metabolites of two soybean lineages (11644 and 13241) submitted to three water conditions: well-watered (WW), water deficit (WD) and rewatered (RW).

|  | *11644* | | | *13241* | | |
| --- | --- | --- | --- | --- | --- | --- |
| *ORGANIC ACIDS* | **WW** | **WD** | **RW** | **WW** | **WD** | **RW** |
| 2-*oxo*-glutarate | 0,01Ab | 0,06Aa | 0,04Ab | 0,10ABa | 0,08Ba | 0,16Aa |
| Ascorbate | 0,08Aa | 0,10Aa | 0,02Bb | 0,01Bb | 0,11Aa | 0,08Aa |
| Citrate | 0,04Aa | 0,05Aa | 0,003Ab | 0,09Ba | 0,05Ba | 0,19Aa |
| *cis*-aconitate | 0.07Ba | 0.08Bb | 1.40Aa | 0.10Ba | 0.79Aa | 0.08Bb |
| Dehydroascorbate | 0,02Ab | 0,05Ab | 0,14Aa | 0,35Aa | 0,20Ba | 0,03Cb |
| Dehydroshikimic acid | 0,01Ab | 0,004Aa | 0,07Aa | 0,19Aa | 0,02Ba | 0,08Ba |
| Fumarate | 0,09Bb | 0,005Cb | 0,13Aa | 0,14Aa | 0,07Ba | 0,07Bb |
| Gluconate | 0,06Aa | 0,16Aa | 0,12Aa | 0,13Aa | 0,08ABa | 0,01Bb |
| Isocitrate | 0,02Cb | 0,09Ba | 0,14Aa | 0,13Aa | 0,10Aa | 0,09Ab |
| Malonate | 0,10Ba | 0,07Ca | 0,12Aa | 0,09Aa | 0,04Bb | 0,06Bb |
| Oxalacetate | 0,07Bb | 0,09Aa | 0,10Aa | 0,09Aa | 0,10Aa | 0,07Bb |
| Phosphoenolpyruvate | 0,04Ba | 0,38Aa | 0,09Bb | 0,05Ba | 0,05Bb | 0,26Aa |
| Succinate | 0,03Bb | 0,09Ab | 0,05Ab | 0,05Ba | 0,08Aa | 0,09Aa |
| Threonate | 0,06Aa | 0,05Ab | 0,04Ab | 0,06Ba | 0,90Aa | 0,96Aa |
| *trans*-caffeic acid | 0,04Bb | 0,10Ab | 0,06Bb | 0,15Ba | 0,24Aa | 0,16Ba |
| *AMINO ACIDS* |  |  |  |  |  |  |
| Alanine | 0,05Ba | 0,09Aa | 0,08ABa | 0,07Ba | 0,10Aa | 0,08ABa |
| Asparagine | 0,09Aa | 0,06Ab | 0,10Ab | 0,06Ba | 0,18Ba | 0,44Aa |
| β-alanine | 0,02Bb | 0,13Aa | 0,03Bb | 0,05Ba | 0,07Bb | 0,26Aa |
| Cysteine | 0,06Ba | 0,15Aa | 0,16Aa | 0,06Ba | 0,13Aa | 0,05Bb |
| Glutamine | 0,02Bb | 0,13Ab | 0,04Bb | 0,06Ba | 0,16Aa | 0,09Ba |
| Glutamate | 0,09Ba | 0,17Aa | 0,06Ba | 0,04Ba | 0,14Aa | 0,07Ba |
| Histidine | 0,09Aa | 0,10Aa | 0,09Aa | 0,38Aa | 0,12Aa | 0,09Aa |
| Isoleucine | 0,06Ba | 0,74Aa | 0,05Bb | 0,03Ca | 0,78Aa | 0,16Ba |
| Leucine | 0,08Bb | 0,99Ab | 0,06Bb | 0,05Ba | 1,09Aa | 0,14Ba |
| Ornithine | 0,07Ba | 0,27Aa | 0,08Ba | 0,08Ba | 0,17Aa | 0,07Ba |
| Valine | 0,08Ba | 0,60Aa | 0,09Ba | 0,05Bb | 0,60Ab | 0,07Bb |
| *SUGARS AND POLYOLS* |  |  |  |  |  |  |
| Erythritol | 0,03Bb | 0,17Ab | 0,06ABb | 0,07Ba | 0,35Aa | 0,31ABa |
| Fructose | 0,09Ba | 0,12ABb | 0,18Aa | 0,13Ba | 0,20Aa | 0,06Cb |
| Fucose | 0,04Ba | 0,08Aa | 0,10Aa | 0,06Ba | 0,09Aa | 0,10Aa |
| Galactitol | 0,07Ba | 0,13Aa | 0,10Ba | 0,07Ba | 0,11Aa | 0,09Ba |
| Galactose | 0,05ABa | 0,02Bb | 0,08Aa | 0,03Ba | 0,10Aa | 0,08Aa |
| Maltose | 0,04Ba | 0,17Aa | 0,14ABa | 0,08Ba | 0,17Aa | 0,11ABb |
| Mannose | 0,004Bb | 0,03Bb | 0,12Aa | 0,06Ba | 0,14Aa | 0,11ABa |
| Inositol | 0,10Aa | 0,03Bb | 0,07ABb | 0,11Ba | 0,22Aa | 0,12Ba |
| Sorbitol | 0,07Ba | 0,07Bb | 0,10Aa | 0,08Aa | 0,11Aa | 0,09Aa |
| Trehalose | 1,00Aa | 0,51Ba | 0,09Ca | 0,07Ab | 0,09Ab | 0,06Aa |
| Xylitol | 0,38Aa | 0,02Aa | 0,03Ab | 0,05Ba | 0,03Ba | 1,05Aa |
| Xylose | 0,01Aa | 0,04Aa | 0,01Ab | 0,01Ba | 0,04Ba | 0,43Aa |
| *POLYAMINES* |  |  |  |  |  |  |
| Cadaverine | 0,01Ba | 0,68Aa | 0,02Bb | 0,03Ba | 0,48ABa | 0,70Aa |
| Putrescine | 0,01Ba | 0,53Aa | 0,14ABa | 0,03Ba | 0,27Aa | 0,28ABa |
| Spermidine | 0,08Ba | 0,13Aa | 0,09Ba | 0,08Ba | 0,16Aa | 0,08Ba |
| Spermine | 0,08Ba | 0,14Ab | 0,08Ba | 0,09Ba | 0,21Aa | 0,06Ba |

Uppercase letters compare the same soybean lineage among water treatments; lowercase letters compare the two soybean lineages for the same water treatment. Different letters denote statistical difference, according to the Tukey test (*p* ≤ 0.05). Degrees of freedom= 17. Data are mean of n = *3* ± *SEM*.

**Table S3.** P-values of statistical results for metabolite data presented in Supplementary Table 2 (Table S2).

| **Metabolite** | ***P-*values** | | |
| --- | --- | --- | --- |
|  | *Water treatment* | *Soybean lineage* | *Water treatment* Soybean lineage* |
| **2-*oxo*-glutarate** | 0.043980 | 0.000082 | 0.022209 |
| **Ascorbate** | 0.00031 | 0.88726 | 0.00126 |
| **Citrate** | 0.227398 | 0.002017 | 0.008323 |
| ***cis*-aconitate** | 0.000445 | 0.059470 | 0.000005 |
| **Dehydroascorbate** | 0.038360 | 0.000821 | 0.000127 |
| **Dehydroshikimic acid** | 0.00091425 | 0.00051239 | 0.00087572 |
| **Fumarate** | 0.000016 | 0.037615 | 0.000091 |
| **Gluconate** | 0.198977 | 0.146057 | 0.016398 |
| **Isocitrate** | 0.017648 | 0.067564 | 0.000120 |
| **Malonate** | 1.973^-05^ | 8.650^-06^ | 1.814^-03^ |
| **Oxalacetate** | 0.05034 | 0.52337 | 0.00150 |
| **Phosphoenolpyruvate** | 0.000002 | 0.005910 | 0.000000 |
| **Succinate** | 0.000598 | 0.035039 | 0.074365 |
| **Threonate** | 3.9841^-06^ | 1.4800^-08^ | 2.6648^-06^ |
| ***trans*-caffeic acid** | 0.000125 | 0.000000 | 0.214750 |
| **Alanine** | 0.01816 | 0.14356 | 0.65738 |
| **Asparagine** | 0.00043766 | 0.00042185 | 0.00104336 |
| **β-alanine** | 1.9774^-07^ | 7.2015^-07^ | 4.5500^-09^ |
| **Cysteine** | 0.0029486 | 0.0084061 | 0.0206331 |
| **Glutamine** | 0.00002 | 0.00570 | 0.64930 |
| **Glutamate** | 0.000088 | 0.076835 | 0.135133 |
| **Histidine** | 0.39963 | 0.29453 | 0.39241 |
| **Isoleucine** | 0.000000 | 0.035883 | 0.008893 |
| **Leucine** | 0.000000 | 0.045870 | 0.068769 |
| **Ornithine** | 0.000101 | 0.128381 | 0.105655 |
| **Valine** | 0.000000 | 0.017138 | 0.075545 |
| **Erythritol** | 0.04429 | 0.02274 | 0.34710 |
| **Fructose** | 0.02756 | 0.95634 | 0.00014 |
| **Fucose** | 0.00031 | 0.21682 | 0.93366 |
| **Galactitol** | 0.000326 | 0.252613 | 0.307982 |
| **Galactose** | 0.011633 | 0.050071 | 0.001549 |
| **Maltose** | 0.00595 | 0.99322 | 0.38407 |
| **Mannose** | 0.00020710 | 0.00060786 | 0.00181651 |
| **Inositol** | 0.173692 | 0.000009 | 0.000088 |
| **Sorbitol** | 0.053225 | 0.029237 | 0.007838 |
| **Trehalose** | 4.7905^-07^ | 1.3080^-08^ | 6.7843^-07^ |
| **Xylitol** | 0.004060 | 0.036941 | 0.000333 |
| **Xylose** | 0.035042 | 0.046725 | 0.024665 |
| **Cadaverine** | 0.007518 | 0.183246 | 0.025209 |
| **Putrescine** | 0.00858 | 0.64431 | 0.15341 |
| **Spermidine** | 0.00196 | 0.46542 | 0.55933 |
| **Spermine** | 0.000006 | 0.072529 | 0.018267 |
