# Supplementary figures and images for: Metabolic adjustment and regulation of gene expression are essential for increased resistance to severe water deficit and resilience post-stress in soybean
Source: PeerJ. 2022 Mar 18;10:e13118. doi: 10.7717/peerj.13118 (PMC8935993; doi:10.7717/peerj.13118)

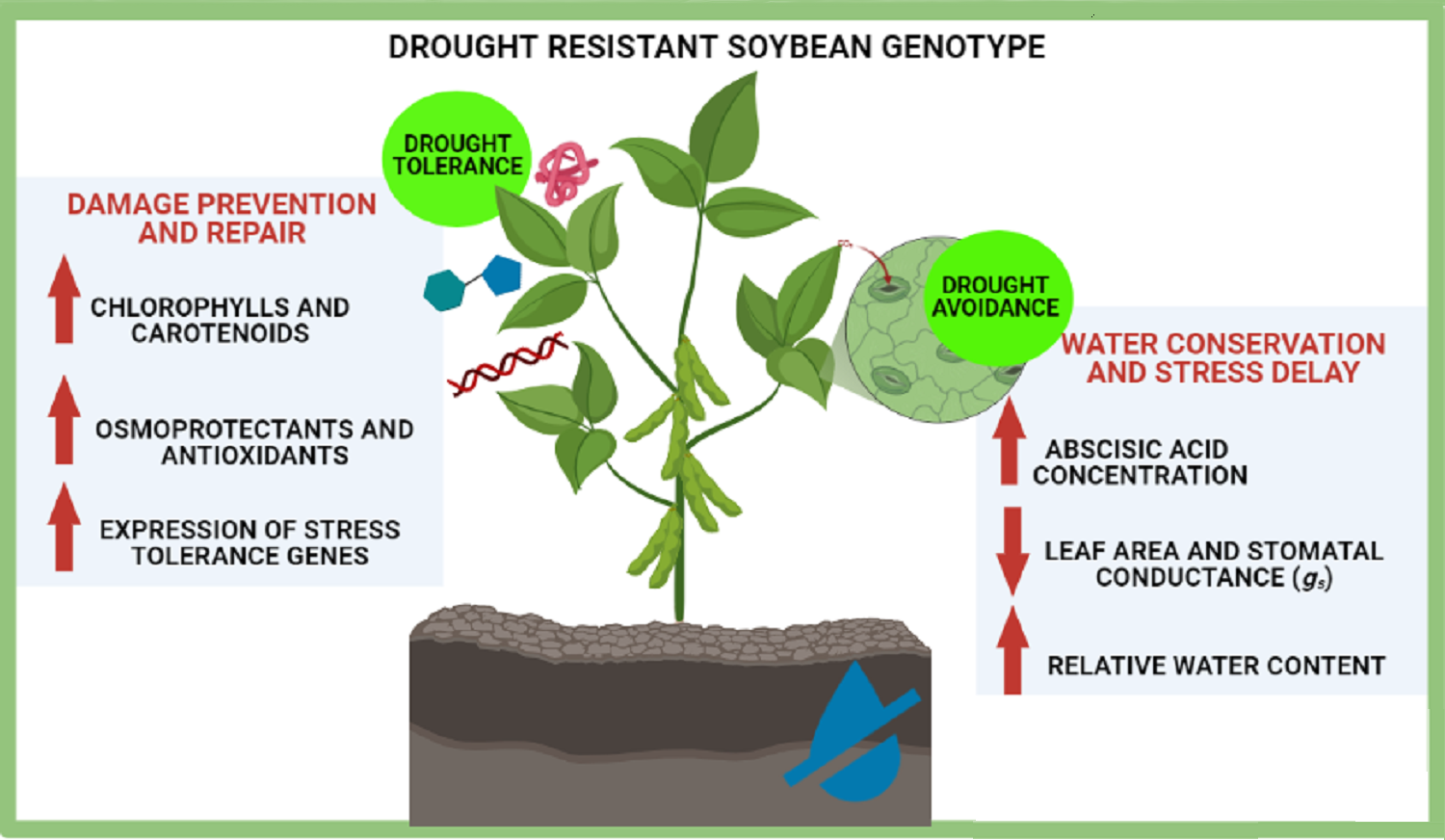

Supplement: Supplemental Information 3 [file peerj-10-13118-s003.png]
